# Gastroesophageal reflux disease and childhood asthma: A bidirectional two-sample Mendelian randomization study

**DOI:** 10.1097/MD.0000000000048171

**Published:** 2026-04-10

**Authors:** Lianfu Ding, Lijuan Xiong, Qingfa Chen, Hong Liu, Qing Li

**Affiliations:** a Department of Emergency, The Affiliated Children’s Hospital of Nanchang Medical College (Jiangxi Provincial Children’s Hospital), Nanchang, Jiangxi, China; b Department of Hematology, The Affiliated Children’s Hospital of Nanchang Medical College (Jiangxi Provincial Children’s Hospital), Nanchang, Jiangxi, China.

**Keywords:** causality, childhood asthma, gastroesophageal reflux, genetic epidemiology, Mendelian randomization

## Abstract

Gastroesophageal reflux disease (GERD) commonly coexists with childhood asthma, but observational evidence is confounded by adiposity, medications, and shared environments, and reverse causation cannot be excluded. Mendelian randomization (MR), using germline variants as instruments, can mitigate confounding and reverse causation. We conducted a bidirectional two-sample MR to clarify whether genetically proxied GERD liability causally increases childhood asthma risk and whether childhood asthma liability influences GERD. Summary-level genome-wide association study (GWAS) data for gastroesophageal reflux and childhood asthma were obtained from the OpenGWAS repository, and both GWAS were conducted in individuals of European ancestry, so the causal estimates are mainly applicable to European populations. Instrumental variables were selected according to standard two-sample MR criteria. Bidirectional MR analyses were performed in R using the TwoSampleMR package. Of 5 complementary MR methods, inverse-variance weighting (IVW) was prespecified as the primary analysis. Heterogeneity was assessed with Cochran *Q* under IVW and MR-Egger models. Robustness was evaluated by leave-one-out analysis, Mendelian Randomization Pleiotropy RESidual Sum and Outlier, and the MR-Egger intercept (for directional pleiotropy). In the forward direction (gastroesophageal reflux → childhood asthma), IVW indicated increased risk (odds ratio = 1.792; 95% confidence interval, 1.661–1.933; *P* = 2.033 × 10^−51^ < .001). Results were consistent across the other 4 MR methods. There was no significant heterogeneity by Cochran *Q*, no evidence of directional pleiotropy by the MR-Egger intercept, no outliers by Mendelian Randomization Pleiotropy RESidual Sum and Outlier, and leave-one-out analysis supported stability. In the reverse direction (childhood asthma → gastroesophageal reflux), IVW showed no association (odds ratio = 1.029; 95% confidence interval, 0.983–1.078; *P* = .225 > .05), with concordant findings from the other MR methods and similarly negative sensitivity tests. Genetic evidence supports a positive causal effect of gastroesophageal reflux on the risk of childhood asthma, whereas childhood asthma does not appear to causally influence gastroesophageal reflux.

## 1. Introduction

Childhood asthma is a common chronic respiratory disorder of individuals typically younger than 18 years, characterized by persistent airway inflammation, variable airflow limitation, and bronchial hyperresponsiveness. Clinical manifestations are heterogeneous, most commonly recurrent wheeze, cough, chest tightness, and dyspnea.^[1,2]^ Compared with adult-onset asthma, childhood asthma often shows distinct phenotypes, environmental triggers, and developmental trajectories, and early-life disease can have long-term consequences for lung growth and lung function across the life course.^[3,4]^ Epidemiologic studies have suggested an association between gastroesophageal reflux disease (GERD) and childhood asthma, but causality remains uncertain.^[5]^ Although many reports link GERD to worsening of asthma symptoms, whether GERD directly causes asthma or exacerbates its symptoms remains debated.^[6]^ Some studies indicate that treating GERD can improve symptoms in a subset of patients with asthma, yet not all patients benefit.^[7]^ The relationship may be bidirectional: GERD may aggravate asthma, while asthma and its treatments (e.g., bronchodilators) may increase the risk of GERD, further complicating interpretation.^[8]^ In the present study, we deliberately focused on childhood asthma rather than asthma in general because GERD is particularly relevant for symptom control and nocturnal exacerbations in pediatric patients, and because the availability of a large, well-characterized childhood asthma genome-wide association study (GWAS) enabled us to target this age-defined phenotype and reduce the heterogeneity that would arise from combining childhood- and adult-onset asthma with potentially different genetic architectures and reflux patterns. Traditional observational designs are vulnerable to residual confounding, and the possibility of two-way causation adds complexity.

Mechanistically, several pathways have been proposed to explain the bidirectional relationship between GERD and asthma.^[9]^ On the GERD-to-asthma axis, refluxed gastric contents (acid, pepsin, and bile salts) may reach the upper and lower airways through micro-aspiration, where pepsin and other components have been detected in bronchoalveolar or airway samples and are thought to promote airway inflammation, bronchial hyper-responsiveness, and chronic cough that can exacerbate asthma symptoms.^[10]^ In addition, esophageal acid exposure can stimulate vagal afferents and trigger reflex bronchoconstriction without overt aspiration, further worsening wheeze and nocturnal symptoms. Conversely, asthma may predispose to GERD through increased negative intrathoracic pressure during bronchospasm, dynamic hyperinflation, and repetitive coughing, which disturb the thoracoabdominal pressure gradient, impair lower esophageal sphincter function, and facilitate transient sphincter relaxations; some bronchodilators may further reduce sphincter tone.^[11]^ Integrative reviews and pediatric studies support this “feedback loop,” showing that asthma increases the risk of GERD and that reflux treatment can improve asthma control in a subset of patients. Recent Mendelian randomization (MR) and genetic correlation studies also suggest a causal effect of GERD on asthma and a partially shared genetic architecture between the 2 diseases, providing biologic support for a complex, bidirectional link.

Childhood asthma is not only a common chronic respiratory disease in children but also a major global public health problem. Recent Global Burden of Disease 2019 analyses indicate that childhood asthma contributes substantial incidence, deaths, and years lived with disability across regions, highlighting a considerable worldwide burden on children and adolescents.^[12]^ At the same time, GERD has emerged as a highly prevalent chronic upper gastrointestinal disorder. A large systematic review estimated that typical reflux symptoms affect around 14% of the global population, and updated GBD 2019 data suggest that nearly 780 million individuals were living with GERD in 2019, with marked increases in prevalence and disability over the past 3 decades.^[13,14]^ Together, these data underscore the substantial global burden of both childhood asthma and GERD and further justify the need to clarify their potential causal relationship.

MR, which uses genetic variants as instrumental variables (IVs), can reduce confounding and reverse causation and has been increasingly applied to causal inference in recent years.^[15–18]^ In this study, we adopted a bidirectional two-sample MR approach to explore the causal relationship between GERD and childhood asthma. Leveraging large GWAS summary data, we tested causal effects in both directions (GERD on asthma and asthma on GERD). This approach helps clarify causality and may offer new perspectives to guide clinical management. The procedures are outlined as follows.

## 2. Materials and methods

### 2.1. Data sources

All summary-level GWAS data used in this study were obtained from the IEU OpenGWAS repository (https://gwas.mrcieu.ac.uk/). Effect estimates for GERD were extracted from a large meta-analysis of GERD (GWAS ID: ebi-a-GCST90000514),^[19]^ in which GERD cases were defined using a broad clinical phenotype based on hospital and primary-care diagnostic codes, endoscopy or medical record data, and self-reported frequent heartburn or acid regurgitation, while participants without GERD-related diagnoses or symptoms served as controls. Summary statistics for childhood asthma were obtained from the European-ancestry pediatric asthma GWAS (GWAS ID: ebi-a-GCST90018895),^[20]^ including 27,712 cases and 411,131 controls; in this dataset, cases were defined as physician-diagnosed childhood-onset asthma occurring in children and adolescents, with onset before adulthood (predominantly < 18 years of age), according to standard clinical criteria across contributing cohorts, and controls were individuals without any diagnosis of asthma. Both GWAS were conducted predominantly in individuals of European ancestry; therefore, the causal estimates primarily reflect genetic architecture in European populations and may not be directly generalizable to other ancestries. Because these meta-analyses were derived from large European population-based cohorts and only summary-level data are available in OpenGWAS, some degree of sample overlap between the GERD and childhood asthma datasets cannot be excluded and could not be formally quantified. The main characteristics of these 2 GWAS datasets are summarized in Table 1.

**Table 1:** Summary of GWAS datasets used in the bidirectional Mendelian randomization analyses.

Trait	GWAS ID	PMID	Phenotype definition/data source	Age range of participants	Sample size (cases/controls; % cases)	Number of SNPs in GWAS	Ancestry breakdown	Variance explained by MR instruments (*R*^2^)*
Gastroesophageal reflux disease (GERD)	ebi-a-GCST90000514	34187846	Broad GERD phenotype based on hospital and primary-care diagnostic codes, endoscopy or medical record data, and self-reported frequent heartburn or acid regurgitation in large European population-based cohorts (e.g., UK Biobank, 23andMe).	Adults from population-based cohorts (predominantly ≥ 18 years; mainly middle-aged and older adults).	129,080/473,524 (21.4% cases)	2,320,781	European ancestry (predominantly White European).	Not reliably quantifiable from available summary statistics; instrument strength summarized by *F*-statistics (all *F* > 10).
Childhood asthma	ebi-a-GCST90018895	34594039	Physician-diagnosed childhood-onset asthma in children and adolescents, with onset predominantly before 18 years of age; controls were individuals without any asthma diagnosis.	Children and adolescents; onset predominantly before 18 years of age.	27,712/411,131 (6.3% cases)	24,166,696	European ancestry (predominantly White European).	0.069 (6.9% of variance in childhood asthma liability explained by the 29-SNP instrument).

GWAS = genome-wide association study, SNP = single-nucleotide polymorphism.

* *R*^2^ refers to the proportion of variance in the exposure explained by the final SNP instrument set used in the Mendelian randomization analyses. For childhood asthma, *R*^2^ was calculated as Σ[2 × EAF × (1 – EAF)×β^2^] based on the 29-SNP instrument. For GERD, *R*^2^ could not be reliably quantified from the available summary statistics, and instrument strength is therefore summarized using *F*-statistics (all *F* > 10).

### 2.2. Instrument selection

Single-nucleotide polymorphisms (SNPs) are strongly associated with the exposures (gastroesophageal reflux disease and childhood asthma); SNPs are independent of any confounders; and SNPs influence the outcomes only through the exposures, without pathways involving other confounders.^[21,22]^ To satisfy these assumptions, SNPs were selected at the genome-wide significance threshold (*P* < 5 × 10^‐08^) and clumped to avoid linkage disequilibrium (LD) (*r*^2^ < 0.001, window = 10,000 kb). In addition, to avoid weak instruments we required an *F*-statistic > 10. The *F*-statistic was calculated as $F=\frac{N-K-1}{K}\times \frac{R^{2}}{1-R^{2}}$, where N is the sample size of the exposure GWAS, *K* is the number of included SNPs, and *R*^2^ is the proportion of variance in the exposure explained by these SNPs. For a single SNP, *R*^2^ = 2 (1‐EAF) × EAF × β^2^, where EAF is the effect-allele frequency and β is the per-allele effect size. During harmonization, palindromic SNPs (A/T or C/G) with intermediate effect allele frequencies (0.42 ≤ EAF ≤ 0.58 in either the exposure or outcome GWAS) were considered strand-ambiguous and were removed, whereas palindromic SNPs with clearly low or high allele frequencies (EAF < 0.42 or >0.58) were retained because strand alignment could be inferred unambiguously. In addition, we further inspected the SNP–outcome associations and influence diagnostics and excluded 13 GERD-associated SNPs that were flagged as potentially pleiotropic or influential (e.g., variants showing outlying or unexpectedly strong direct associations with childhood asthma relative to the remaining instruments). To improve transparency, these excluded SNPs are summarized in 2 supplementary tables: Table S1, Supplemental Digital Content, https://links.lww.com/MD/R657 lists their alleles, effect allele frequencies and association estimates for GERD (exposure) and childhood asthma (outcome), and Table S2, Supplemental Digital Content, https://links.lww.com/MD/R657 provides, for each excluded SNP, the nearest or reported gene/region, a brief summary of problematic associations reported in previous GWAS/MR studies, and the rationale for exclusion from the final instrument set. Finally, we used LDtrait (https://ldlink.nih.gov/?tab=ldtrait) to examine the included SNPs to ensure they did not violate assumptions (2) and (3), and applied Mendelian Randomization Pleiotropy RESidual Sum and Outlier (MR-PRESSO) to test for potential outlier SNPs indicative of horizontal pleiotropy.

### 2.3. MR analyses

In this bidirectional MR design, the forward analysis treated gastroesophageal reflux as the exposure and childhood asthma as the outcome; the reverse analysis treated childhood asthma as the exposure and gastroesophageal reflux as the outcome. Two-sample MR was performed using 5 complementary estimators: inverse-variance weighting (IVW), MR-Egger, weighted median (WM), simple mode (SM), and weighted mode (WMo). The IVW estimate was prespecified as the primary result. Heterogeneity was assessed with Cochran *Q* statistic. Horizontal pleiotropy was evaluated via MR-Egger regression, focusing on the MR-Egger intercept (an intercept not different from zero indicates no directional pleiotropy). Sensitivity was examined using leave-one-out analyses, and potential bias was visually inspected with funnel plots.

### 2.4. Sensitivity analyses

Between-instrument heterogeneity was evaluated using Cochran *Q* under IVW and MR-Egger frameworks. Horizontal pleiotropy was assessed by the MR-Egger intercept and the MR-PRESSO global test. When the MR-PRESSO global test indicates significant distortion, outlier-corrected estimates can in principle be obtained by removing the detected outlier SNPs and recomputing the IVW estimate; however, in the present analyses the MR-PRESSO global test did not identify any outlier SNPs in either direction, so MR-PRESSO was used purely as a diagnostic tool and no variants were removed on the basis of MR-PRESSO. Leave-one-out analyses were performed to identify influential variants. Symmetry of funnel plots and consistency across the 5 MR estimators were examined to appraise small-study effects and robustness. Collectively, these procedures were used to verify that the primary IVW results were not driven by heterogeneity, directional pleiotropy, or single-variant influence.

### 2.5. Ethics statement

All analyses used publicly available, de-identified GWAS summary statistics from the IEU OpenGWAS repository. No new individual-level or identifiable human data were collected, and no recruitment or intervention was performed. In accordance with the Declaration of Helsinki and local regulations, this secondary analysis was exempt from institutional review board review and from the requirement for written informed consent. Data use complied with the original GWAS consents and data-access policies.

### 2.6. Statistical analysis

Bidirectional two-sample MR analyses were conducted in R (version 4.4.1; R Foundation for Statistical Computing, Nanchang, Jiangxi Province, China) using TwoSampleMR (v0.5.6) as the main workflow and MR-PRESSO (v1.0) for outlier detection. Five estimators were implemented: inverse-variance weighting (IVW; prespecified primary), MR-Egger, WM, SM, and WMo. For binary outcomes, effect estimates are presented as odds ratios (ORs) with 95% confidence intervals (CIs) per one-unit increase in genetically proxied exposure liability on the log-odds scale. We did not rescale the MR estimates to correspond to a doubling of disease risk. Statistical significance was assessed using two-sided tests with a Bonferroni-corrected threshold of α = 0.025 (0.05/2) to account for testing causal effects in 2 directions (GERD → childhood asthma and childhood asthma → GERD). Forest, scatter, single-SNP, and funnel plots were generated to visualize effect sizes and potential small-study bias. For the reverse direction (childhood asthma as the exposure and GERD as the outcome), we additionally quantified the strength of the 29-SNP instrument. Using the formula *R*^2^ = Σ[2 × EAF × (1 − EAF)×β^2^], these variants jointly explained approximately 6.9% of the variance in childhood asthma liability (*R*^2^ = 0.069), with a corresponding *F*-statistic of 1118, indicating very strong instruments. Based on this *R*^2^, the GERD GWAS sample size (129,080 cases and 473,524 controls), and a Bonferroni-corrected significance level of α = 0.025, a post hoc MR power calculation (mRnd) showed essentially complete power (≈100%) to detect clinically modest causal effects of childhood asthma on GERD (e.g., odds ratio ≥ 1.10). Thus, the null result in the reverse direction is unlikely to be explained by insufficient statistical power.

## 3. Results

### 3.1. Two-sample MR results (gastroesophageal reflux as the exposure; childhood asthma as the outcome)

#### 3.1.1. Instrument selection

**Figure 1.**
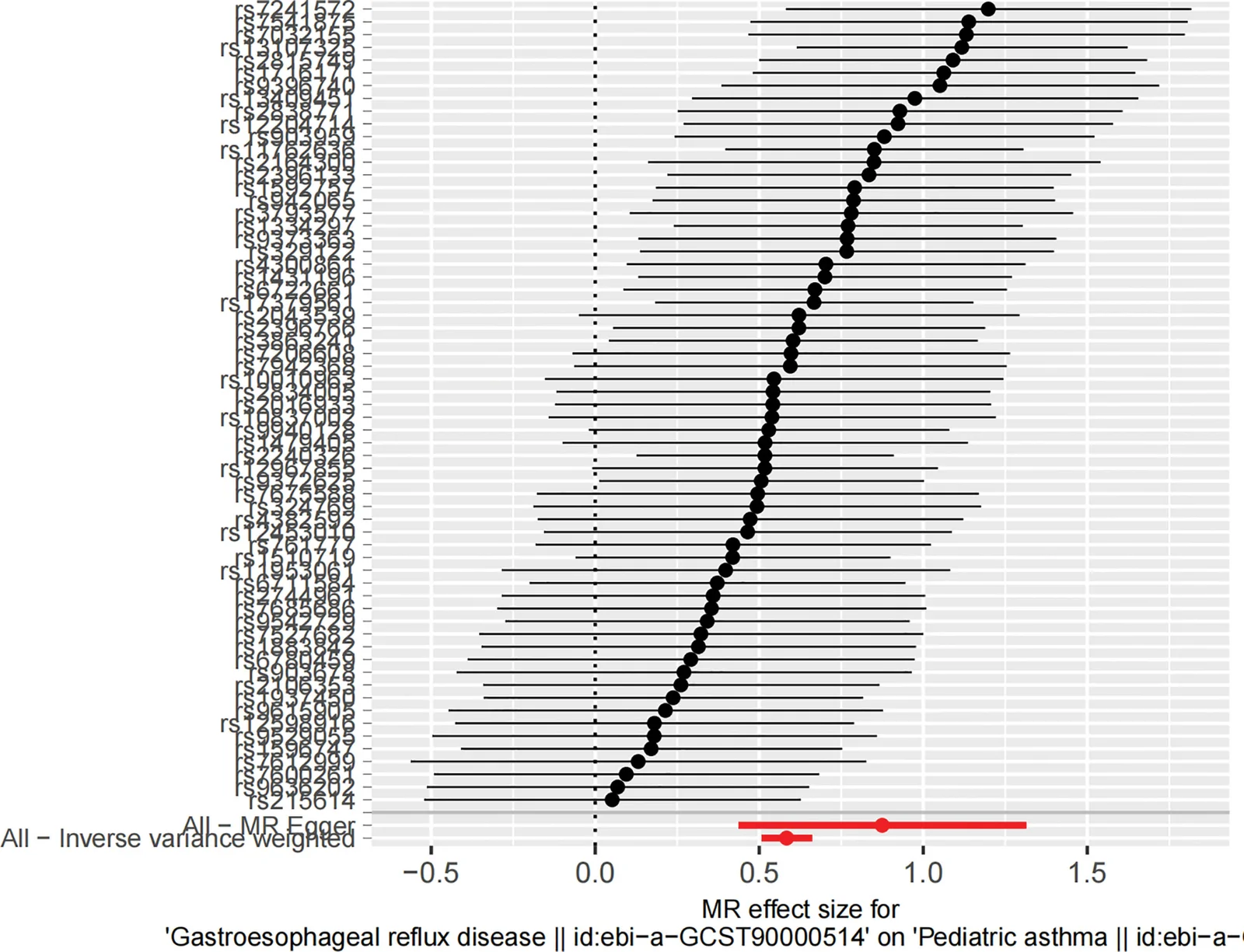
Forest plot of Mendelian randomization (MR) analysis with gastroesophageal reflux disease (GERD) as the exposure.

When GERD was treated as the exposure and childhood asthma as the outcome, 80 SNPs were initially identified. After excluding 13 SNPs using LDtrait (https://ldlink.nih.gov/?tab=ldtrait), 67 SNPs remained as IVs. LDtrait screening indicated that these 13 SNPs were associated with traits such as adiposity and anthropometric measures, cardiometabolic diseases, neuropsychiatric or neurobehavioural traits, immune or autoimmune conditions, and asthma or asthma-related phenotypes, suggesting potential horizontal pleiotropy (Table S2, Supplemental Digital Content, https://links.lww.com/MD/R657). For the forward MR analysis (gastroesophageal reflux disease as the exposure and childhood asthma as the outcome), 67 independent GERD-associated SNPs were selected as IVs after LD clumping and harmonization with the childhood asthma GWAS. All instruments had *F*-statistics >10, indicating that weak instrument bias was unlikely. Detailed SNP-level characteristics (including effect allele, effect-allele frequency, per-allele log-odds ratio, standard error, *P* value, and *F*-statistic for each variant) are provided in Table S3, Supplemental Digital Content, https://links.lww.com/MD/R657. The forest plot derived from them is shown in Figure 1. The 13 excluded SNPs, along with their associations with GERD and childhood asthma, are presented in Table S1, Supplemental Digital Content, https://links.lww.com/MD/R657 and their problematic associations and reasons for exclusion are summarized in Table S2, Supplemental Digital Content, https://links.lww.com/MD/R657.

#### 3.1.2. MR analysis

**Figure 2.**
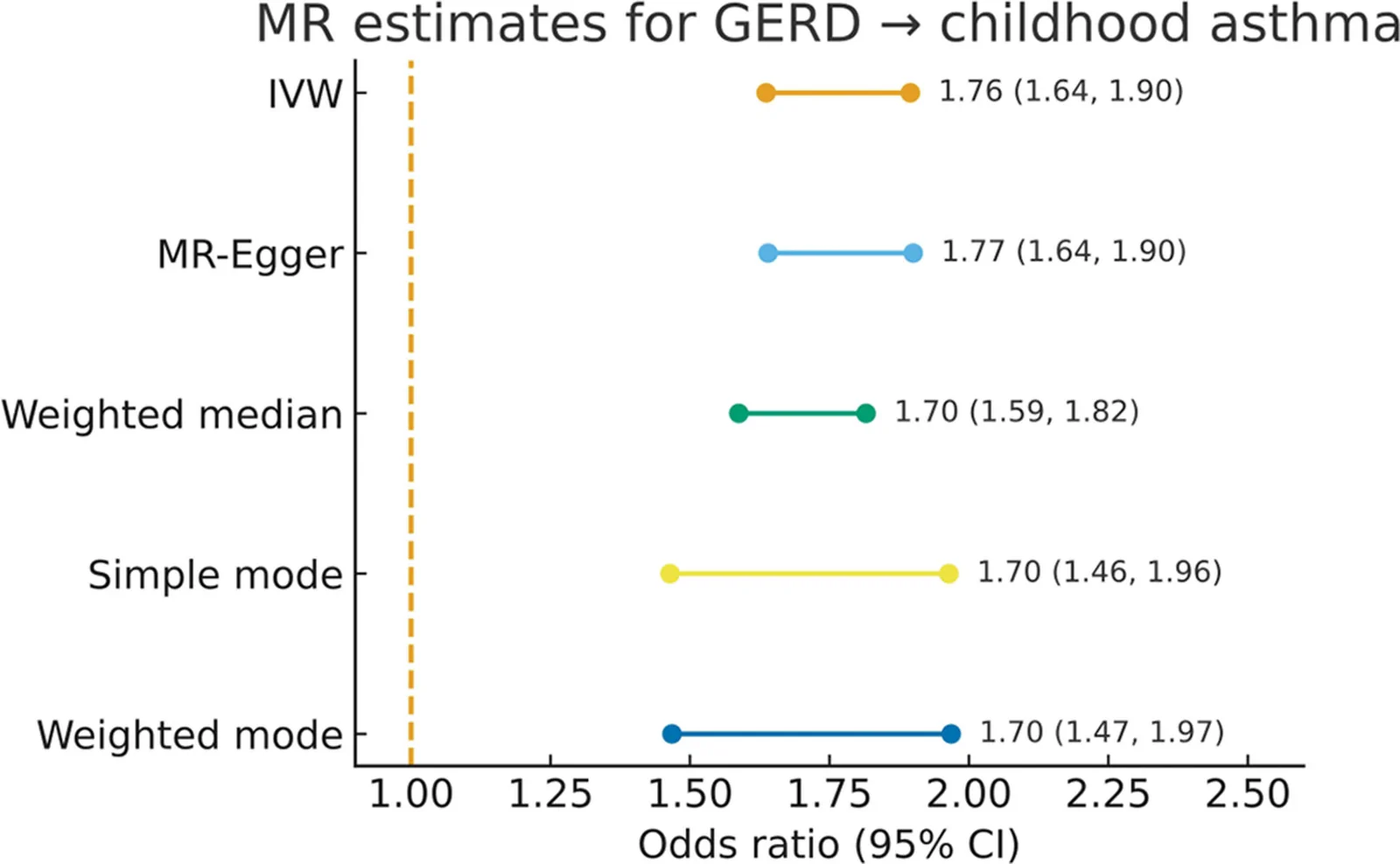
Forest plot of Mendelian randomization (MR) estimates across 5 methods with gastroesophageal reflux disease (GERD) as the exposure.

In the forward direction (GERD → childhood asthma), the overall workflow and key findings are summarized in Figure S1, Supplemental Digital Content, https://links.lww.com/MD/R656. In the forward MR analysis (gastroesophageal reflux as the exposure; childhood asthma as the outcome), the IVW estimate indicated increased risk (OR = 1.792; 95% CI, 1.661–1.933; *P* = 2.033 × 10^−51^ < .001), interpreted as the change in childhood asthma risk per one-unit increase in the log-odds of genetically proxied GERD liability. Results from the other 4 MR methods were consistent with the IVW estimate (Table 2). In addition, Figure 2 presents a forest plot that directly compares the odds ratios and 95% confidence intervals from all 5 MR methods for the GERD → childhood asthma direction.

**Table 2:** Results of forward Mendelian randomization (MR) analysis with gastroesophageal reflux disease (GERD) as the exposure.

Methods	β	SE	OR	95% CI	*P*	*I*^2^ (%)	MR-Egger intercept *P*
IVW	0.583	0.039	1.792	1.661–1.933	2.033 × 10^−51^	0.0	NA
MR-Egger	0.875	0.223	2.398	1.548–3.715	2.286 × 10^−4^	NA	.921
Weighted median	0.522	0.060	1.686	1.499–1.895	2.410 × 10^−18^	NA	NA
Simple mode	0.518	0.141	1.679	1.274–2.213	4.877 × 10^−4^	NA	NA
Weighted mode	0.519	0.132	1.678	1.297–2.174	2.107 × 10^−4^	NA	NA

*I*^2^ (%) is reported only for the IVW model because Cochran *Q* and *I*^2^ are defined for the inverse-variance weighted meta-analytic framework across SNP-specific Wald ratios. The MR-Egger intercept *P* value is shown only for the MR-Egger method, as this statistic tests for directional horizontal pleiotropy in the MR-Egger regression and is not applicable to the other estimators.

CI = confidence interval, IVW = inverse-variance weighted, NA = not applicable, OR = odds ratio, SNP = single-nucleotide polymorphism.

#### 3.1.3. Sensitivity and robustness analyses

**Figure 3.**
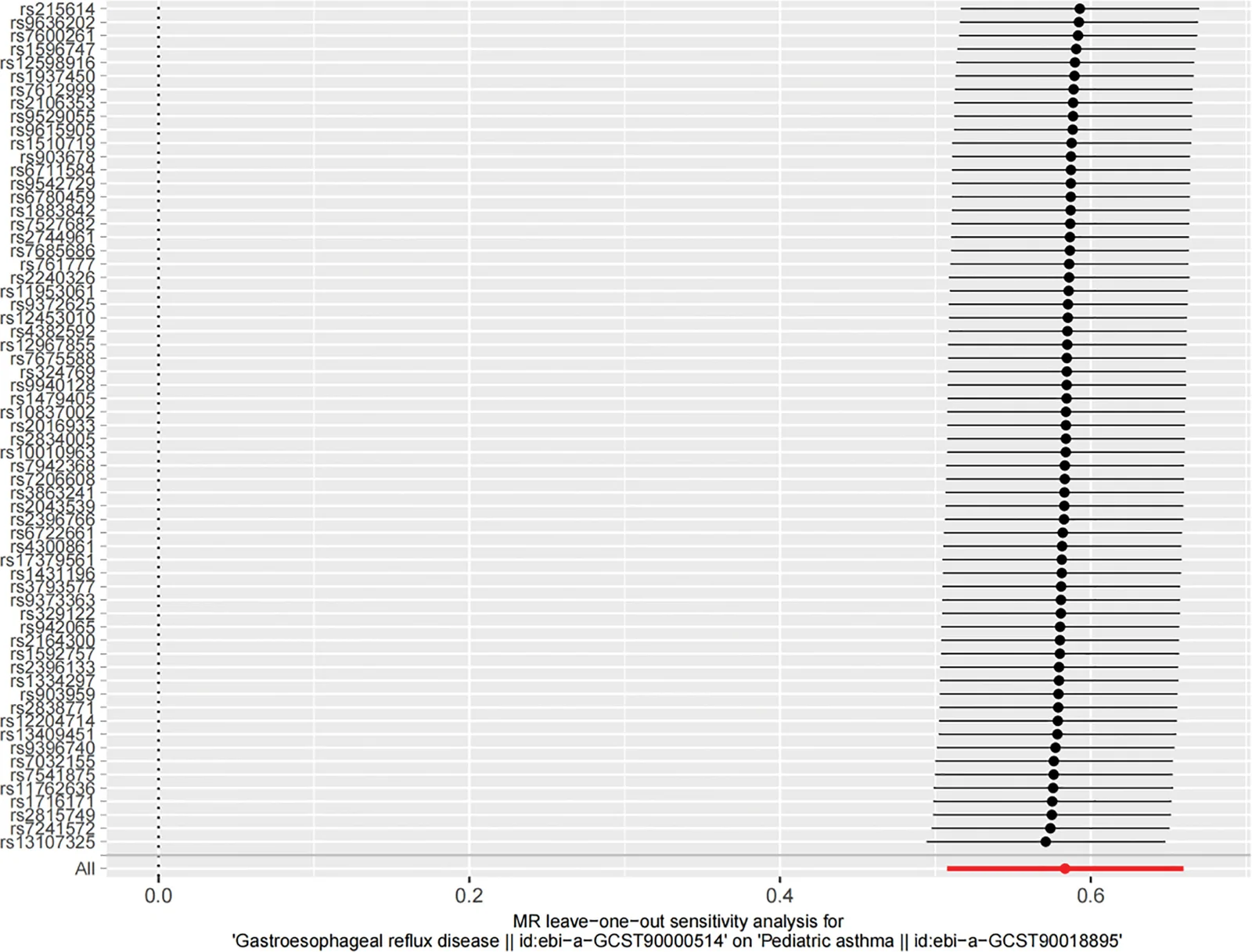
Leave-one-out (LOO) analysis for MR with GERD as the exposure. GERD = gastroesophageal reflux disease, MR = Mendelian randomization.

**Figure 4.**
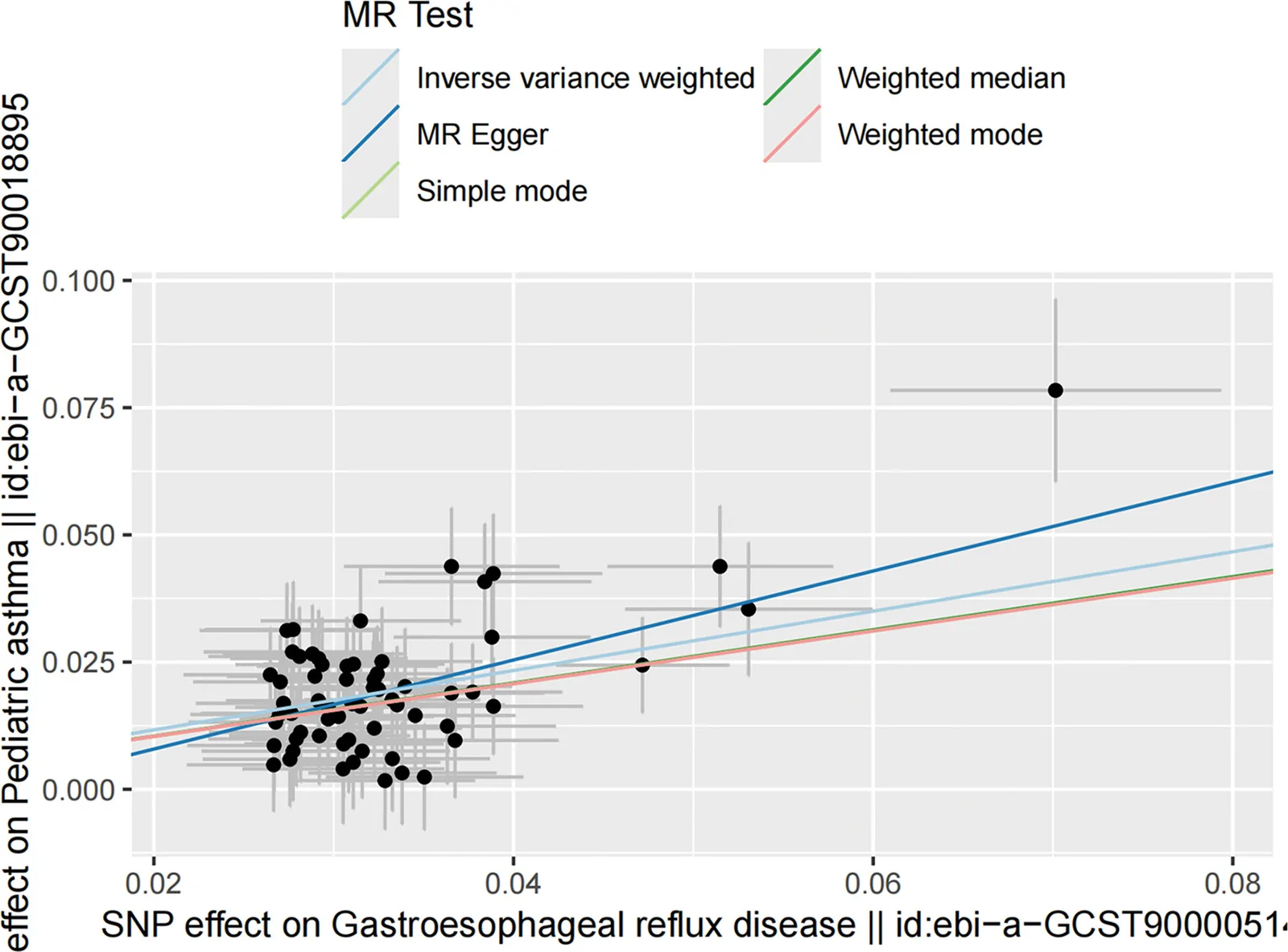
Scatter plot of MR analysis with GERD as the exposure. GERD = gastroesophageal reflux disease, MR = Mendelian randomization.

**Figure 5.**
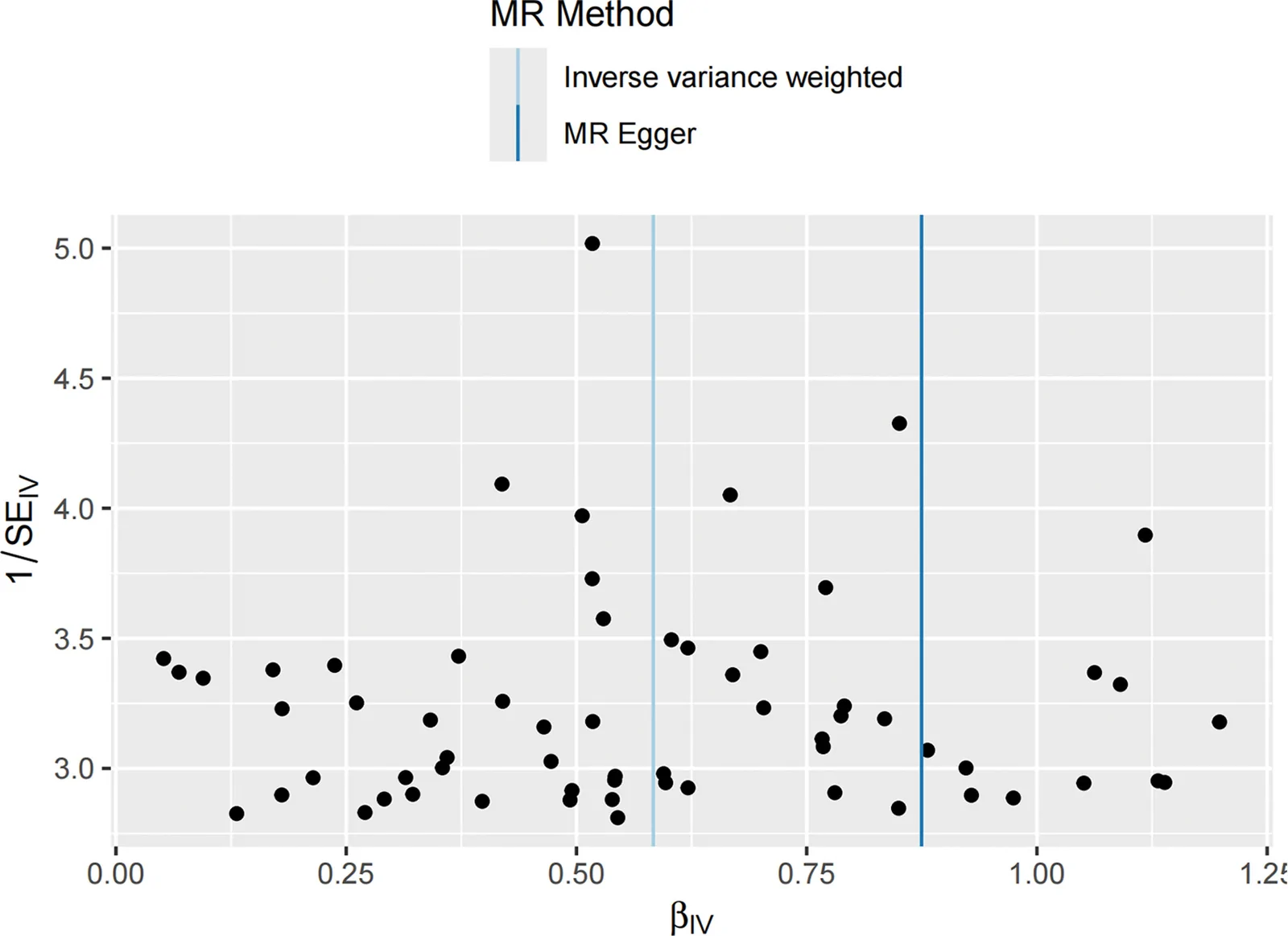
Funnel plot of MR analysis with GERD as the exposure. GERD = gastroesophageal reflux disease, MR = Mendelian randomization.

Under IVW, Cochran *Q* test yielded *P* = .681 (>.05), and under MR-Egger *P* = .708 (>.05), indicating no evidence of heterogeneity. The MR-Egger intercept was 0.009 with *P* = .190, not different from zero, suggesting no horizontal pleiotropy. The MR-PRESSO global test returned *P* = .755, with no outlier SNPs detected. Accordingly, no variants were removed on the basis of MR-PRESSO in the forward analysis. Leave-one-out analyses identified no influential variants (Fig. 3). Scatter plots from the 5 MR estimators showed consistent trends (Fig. 4). The funnel plot was symmetric, arguing against substantial small-study or directional bias (Fig. 5). Collectively, these findings support the reliability and stability of the forward MR results.

### 3.2. Two-sample MR results (childhood asthma as the exposure; gastroesophageal reflux as the outcome)

#### 3.2.1. Instrument selection

**Figure 6.**
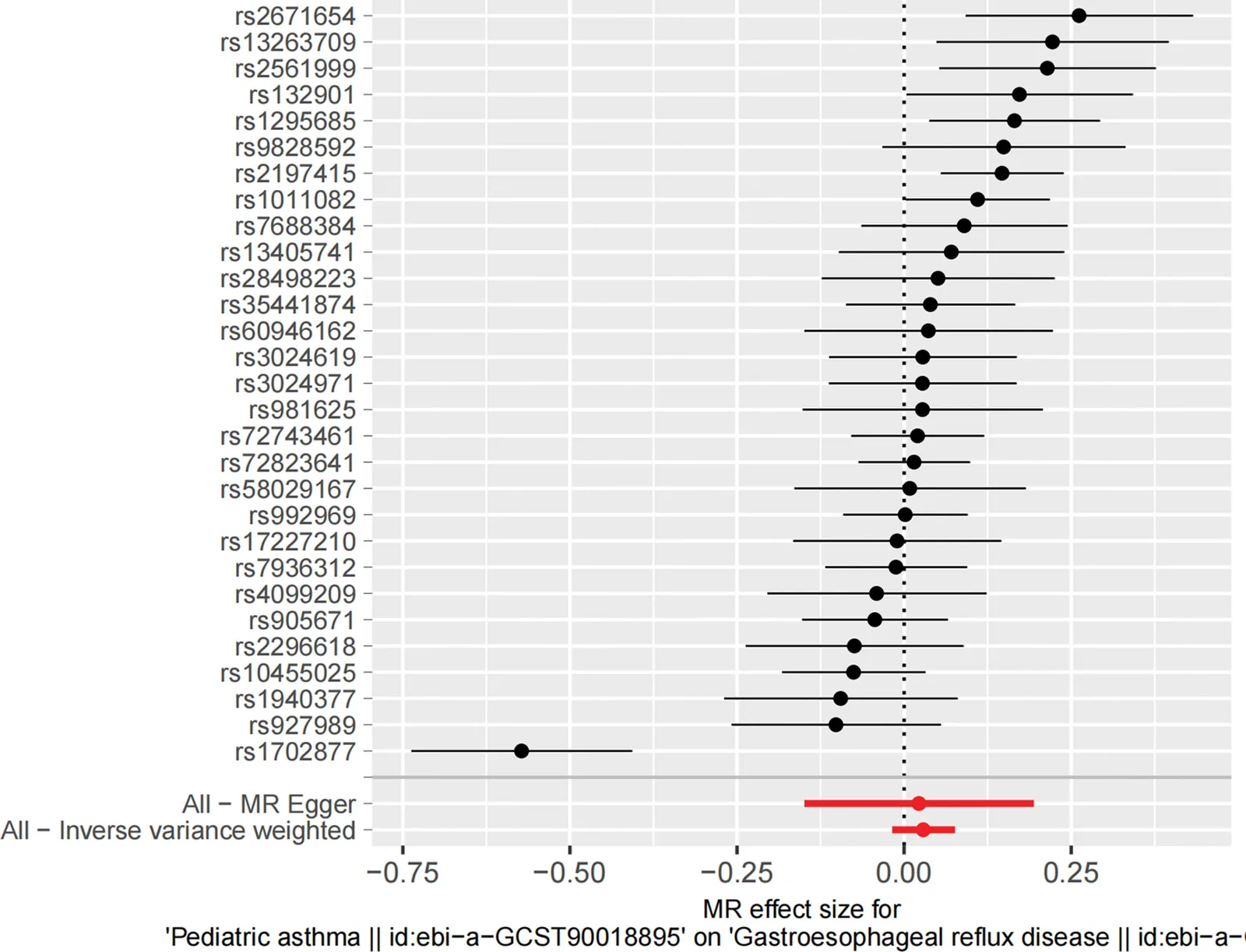
Forest plot of MR analysis with childhood asthma as the exposure. MR = Mendelian randomization.

When childhood asthma was treated as the exposure and GERD as the outcome, 29 SNPs were initially identified. Using LDtrait (https://ldlink.nih.gov/?tab=ldtrait), no SNPs were excluded, leaving 29 SNPs as IVs. Information on the included SNPs is provided in Table 3, and the forest plot based on these SNPs is shown in Figure 6.

**Table 3:** Final included SNPs information (childhood asthma → gastroesophageal reflux).

SNPs	chr	EA	OA	EAF	Childhood asthma			Gastroesophageal reflux			*F*
					β	SE	*P*	β	SE	*P*	
rs1011082	17	C	T	0.543	0.088	9.200 × 10^‐3^	1.355 × 10^‐21^	9.681 × 10^‐3^	4.815 × 10^‐3^	4.436 × 10^‐2^	91.493
rs10455025	5	C	A	0.264	0.092	9.700 × 10^‐3^	2.037 × 10^‐21^	‐6.980 × 10^‐3^	5.022 × 10^‐3^	1.646 × 10^‐1^	89.956
rs1295685	5	G	A	0.759	‐0.097	1.170 × 10^‐2^	1.979 × 10^‐16^	‐1.593 × 10^‐2^	6.270 × 10^‐3^	1.105 × 10^‐2^	68.734
rs13263709	8	C	T	0.591	‐0.057	9.600 × 10^‐3^	3.200 × 10^‐9^	‐1.268 × 10^‐2^	5.043 × 10^‐3^	1.195 × 10^‐2^	35.254
rs132901	22	T	C	0.830	‐0.068	1.130 × 10^‐2^	2.447 × 10^‐9^	‐1.165 × 10^‐2^	5.811 × 10^‐3^	4.495 × 10^‐2^	36.213
rs13405741	2	C	T	0.072	0.094	1.570 × 10^‐2^	1.889e × 10^‐9^	6.664 × 10^‐3^	8.091 × 10^‐3^	4.101 × 10^‐1^	35.847
rs1702877	12	T	C	0.297	0.060	9.700 × 10^‐3^	5.976 × 10^‐10^	‐3.446 × 10^‐2^	5.062 × 10^‐3^	9.9793 × 10^‐12^	38.261
rs17227210	21	T	C	0.144	0.087	1.330 × 10^‐2^	5.981 × 10^‐11^	‐9.126 × 10^‐4^	6.865 × 10^‐3^	8.942 × 10^‐1^	42.789
rs1940377	11	A	G	0.212	‐0.066	1.130 × 10^‐2^	6.130 × 10^–9^	6.215 × 10^‐3^	5.818 × 10^‐3^	2.854 × 10^‐1^	34.114
rs2197415	10	G	T	0.652	0.105	9.400 × 10^‐3^	7.807 × 10^‐29^	1.532E‐02	4.880 × 10^‐3^	1.690 × 10^‐3^	124.774
rs2296618	1	G	A	0.140	‐0.085	1.360 × 10^‐2^	4.074 × 10^‐10^	6.339 × 10^‐3^	7.068 × 10^‐3^	3.698 × 10^‐1^	39.063
rs2561999	2	G	A	0.353	‐0.064	9.900 × 10^‐3^	7.406 × 10^‐11^	‐1.378 × 10^‐2^	5.298 × 10^‐3^	9.299 × 10^‐3^	41.792
rs2671654	17	G	A	0.356	‐0.058	9.400 × 10^‐3^	5.016 × 10^‐10^	‐1.526 × 10^‐2^	5.046 × 10^‐3^	2.486 × 10^‐3^	38.072
rs28498223	14	T	C	0.219	0.061	1.040 × 10^‐2^	5.334 × 10^‐9^	3.075 × 10^‐3^	5.371 × 10^‐3^	5.670 × 10^‐1^	34.403
rs3024619	16	A	G	0.402	0.071	9.700 × 10^‐3^	2.523 × 10^‐13^	1.976 × 10^‐3^	5.052 × 10^‐3^	6.957 × 10^‐1^	53.576
rs3024971	12	G	T	0.077	-0.109	1.530 × 10^-2^	9.861 × 10^-13^	-2.995 × 10^-3^	7.782 × 10^-3^	7.003 × 10^‐1^	50.754
rs35441874	16	A	T	0.200	-0.087	1.080 × 10^-2^	8.870 × 10^-16^	-3.417 × 10^-3^	5.598 × 10^-3^	5.416 × 10^‐1^	64.892
rs4099209	5	G	T	0.181	0.074	1.300 × 10^‐2^	1.242 × 10^‐8^	-3.049 × 10^-3^	6.179 × 10^‐3^	6.217 × 10^‐1^	32.402
rs58029167	9	G	A	0.371	0.061	1.060 × 10^-2^	8.205 × 10^–9^	5.182 × 10^‐4^	5.371 × 10^‐3^	9.231 × 10^‐1^	33.117
rs60946162	3	T	C	0.431	0.051	9.300 × 10^-3^	3.336 × 10^‐8^	1.864 × 10^‐3^	4.853 × 10^‐3^	7.009 × 10^‐1^	30.073
rs72743461	15	A	C	0.188	0.112	1.090 × 10^-2^	8.348 × 10^-25^	2.256 × 10^-3^	5.669 × 10^-3^	6.907 × 10^‐1^	105.58
rs72823641	2	A	T	0.105	‐0.165	1.360 × 10^-2^	1.181 × 10^-33^	^‐^2.449 × 10^‐3^	6.961 × 10^‐3^	7.249 × 10^‐1^	147.194
rs7688384	4	T	C	0.325	‐0.066	9.900 × 10^-3^	2.689 × 10^-11^	-5.930 × 10^-3^	5.171 × 10^-3^	2.514 × 10^-1^	44.444
rs7936312	11	T	G	0.464	0.089	9.300 × 10^-3^	7.976 × 10^-22^	-1.092 × 10^-3^	4.813 × 10^-3^	8.204 × 10^-1^	91.583
rs905671	6	T	A	0.251	-0.091	9.800 × 10^-3^	1.516 × 10^-20^	3.976 × 10^-3^	5.025 × 10^-3^	4.289 × 10^-1^	86.224
rs927989	13	C	G	0.631	0.067	1.010 × 10^-2^	3.624 × 10^-11^	-6.832 × 10^-3^	5.335 × 10^‐3^	2.003 × 10^‐1^	44.005
rs981625	13	G	C	0.055	0.107	1.880 × 10^-2^	1.359 × 10^-8^	2.934 × 10^-3^	9.763 × 10^-3^	7.638 × 10^-1^	32.393
rs9828592	3	C	T	0.432	0.052	9.300 × 10^-3^	1.984 × 10^‐8^	7.772 × 10^‐3^	4.828 × 10^‐3^	1.074 × 10^‐1^	31.264
rs992969	9	G	A	0.810	‐0.117	1.070 × 10^‐2^	9.406 × 10^‐28^	‐1.979 × 10^‐4^	5.536 × 10^‐3^	9.715 × 10^‐1^	119.565

#### 3.2.2. MR analysis

**Figure 7.**
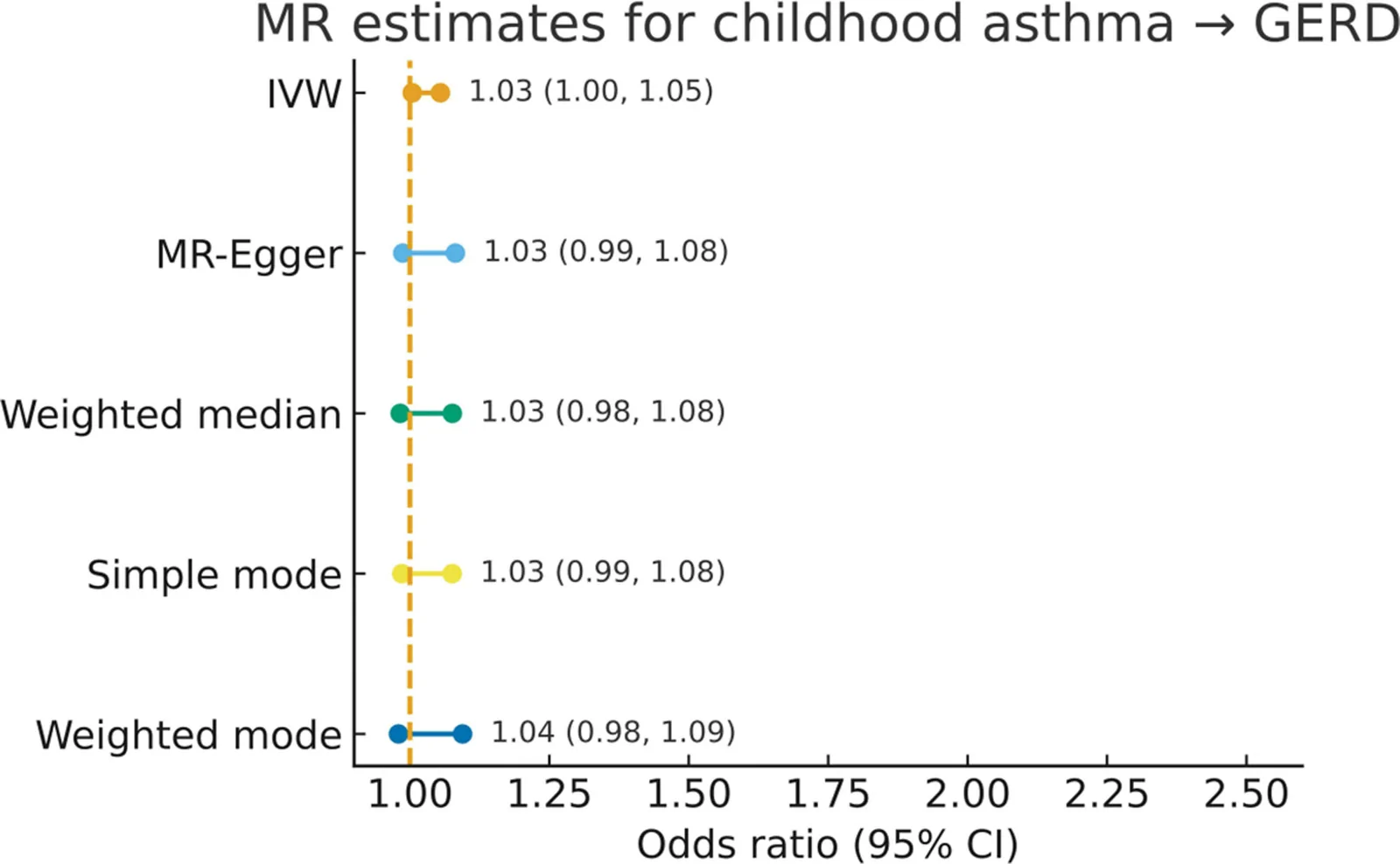
Forest plot of Mendelian randomization (MR) estimates across 5 methods with childhood asthma as the exposure.

In the reverse MR analysis (childhood asthma as the exposure; gastroesophageal reflux as the outcome), the IVW estimate showed no association (OR = 1.029; 95% CI, 0.983–1.078; *P* = .225 > .025). The other 4 MR methods produced concordant null results (Table 4). In addition, a post hoc power analysis based on the 29-SNP childhood asthma instrument (*R*^2^ = 0.069; *F* = 1118) indicated that, given the GERD GWAS sample size and α = 0.025, the study had essentially complete power (≈100%) to detect clinically modest increases in GERD risk (e.g., odds ratio ≥ 1.10). Therefore, the absence of a causal effect from childhood asthma to GERD is unlikely to be attributable to limited statistical power. The other 4 MR methods produced concordant null results (Table 4). Figure 7 provides a corresponding forest plot that displays the side-by-side odds ratios and 95% confidence intervals from all 5 MR estimators for the childhood asthma → GERD direction.

**Table 4:** Results of reverse Mendelian randomization (MR) analysis with childhood asthma as the exposure.

Methods	β	SE	OR	95% CI	*P*	*I*^2^ (%)	MR-Egger intercept *P*
IVW	0.029	0.024	1.029	0.983–1.078	.225	72.1	NA
MR-Egger	0.022	0.087	1.022	0.862–1.213	.801	NA	.142
Weighted median	0.020	0.019	1.020	0.982–1.059	.310	NA	NA
Simple mode	0.018	0.035	1.018	0.950–1.091	.613	NA	NA
Weighted mode	0.012	0.030	1.012	0.955–1.072	.698	NA	NA

*I*^2^ (%) is reported only for the IVW model because Cochran *Q* and *I*^2^ are defined for the inverse-variance weighted meta-analytic framework across SNP-specific Wald ratios. The MR-Egger intercept *P* value is shown only for the MR-Egger method, as this statistic tests for directional horizontal pleiotropy in the MR-Egger regression and is not applicable to the other estimators.

CI = confidence interval, IVW = inverse-variance weighted, NA = not applicable, OR = odds ratio, SNP = single-nucleotide polymorphism.

#### 3.2.3. Sensitivity and robustness analyses

**Figure 8.**
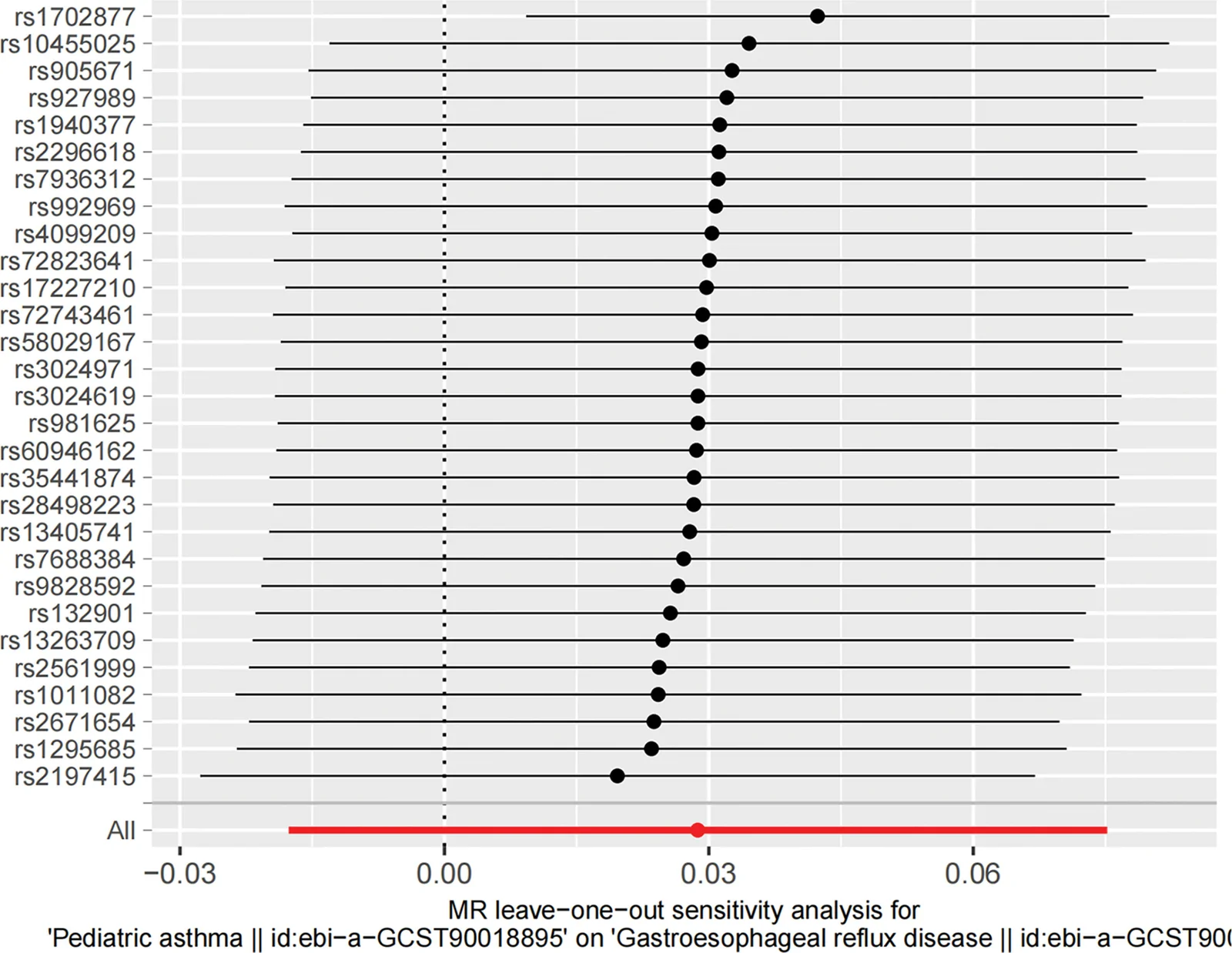
Leave-one-out (LOO) analysis for MR with childhood asthma as the exposure. MR = Mendelian randomization.

**Figure 9.**
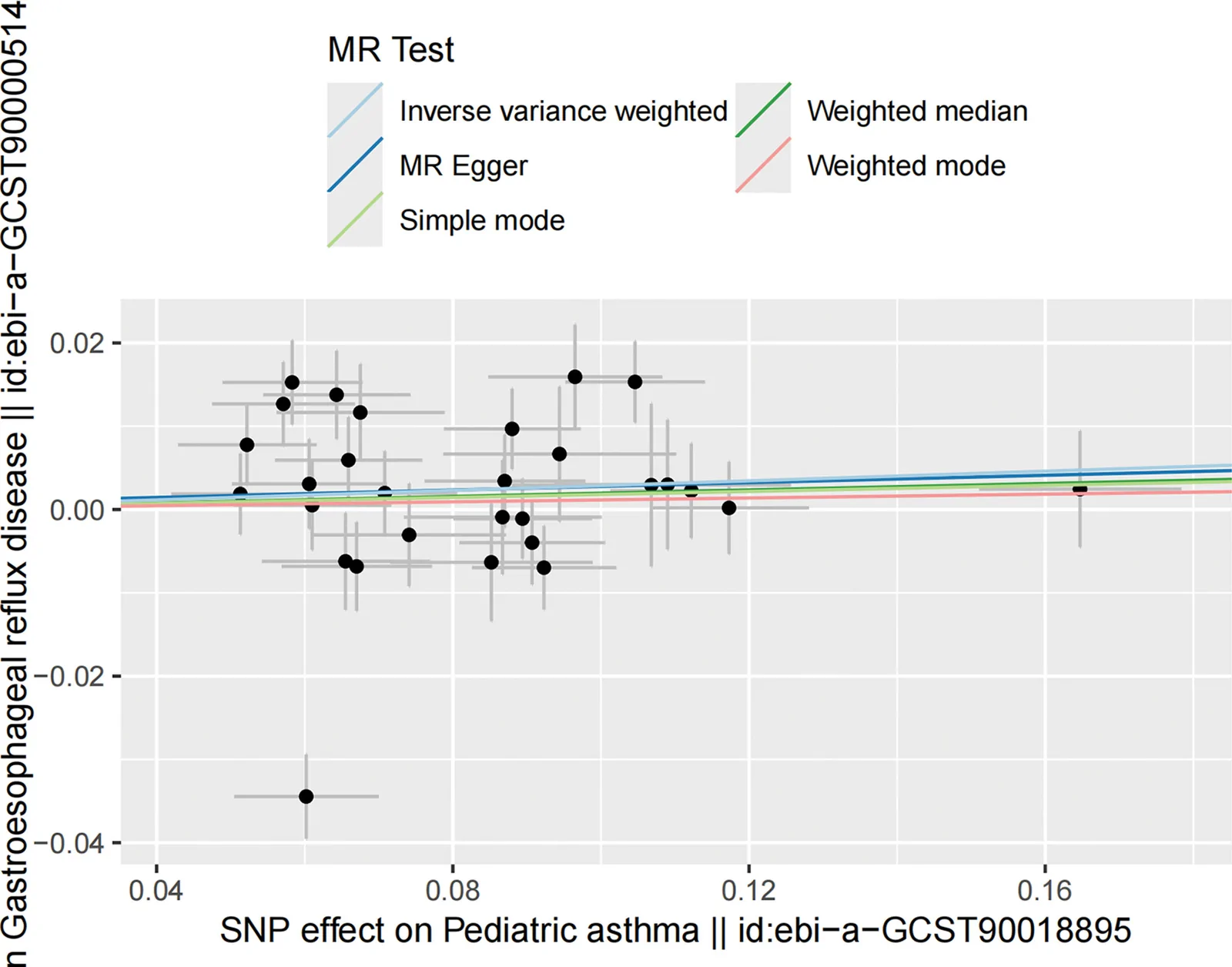
Scatter plot of MR analysis with childhood asthma as the exposure. MR = Mendelian randomization.

**Figure 10.**
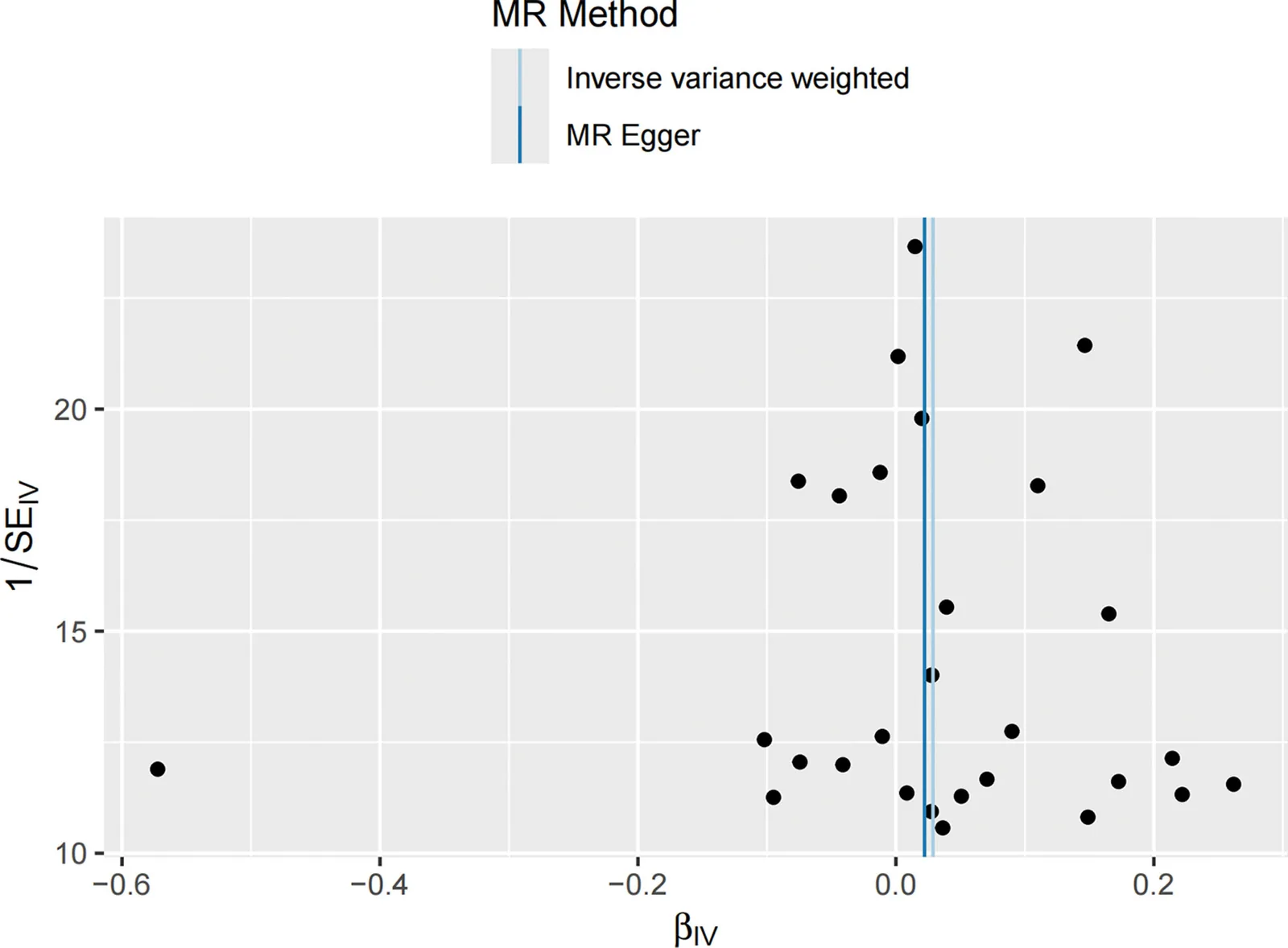
Funnel plot of MR analysis with childhood asthma as the exposure. MR = Mendelian randomization.

Under IVW, Cochran *Q* test yielded *P* = .463 (>.05), and under MR-Egger *P* = .237 > .05), indicating no evidence of heterogeneity in the causal effect of childhood asthma on gastroesophageal reflux. The MR-Egger intercept was 0.0005 with *P* = .939, not different from zero, suggesting no horizontal pleiotropy. The MR-PRESSO global test returned *P* = .559, with no outlier SNPs detected. Thus, no SNPs were removed based on MR-PRESSO in the reverse analysis. Leave-one-out analyses identified no influential variants (Fig. 8). Scatter plots from the 5 MR estimators showed consistent trends (Fig. 9). The funnel plot was symmetric, arguing against substantial small-study or directional bias (Fig. 10). Together, these findings support the reliability and stability of the reverse MR results.

## 4. Discussion

GERD is defined as the reflux of gastric contents into the esophagus that leads to troublesome symptoms and/or complications.^[13]^ Its clinical manifestations include heartburn, acid regurgitation, and chest pain. Childhood asthma is a common chronic respiratory disease characterized by persistent airway inflammation and hyperresponsiveness, typically presenting with recurrent wheeze, cough, chest tightness, and dyspnea.^[23]^ Epidemiologic research has suggested an association between GERD and childhood asthma.^[24]^ Multiple observational studies report a higher prevalence of GERD among patients with asthma, and, conversely, a higher prevalence of asthma among patients with GERD.^[25–27]^ Mechanistically, GERD may exacerbate asthma by acid reflux irritating the airways, triggering reflex bronchospasm, or increasing airway hyperresponsiveness.^[28]^ In the opposite direction, asthma and its treatments (e.g., bronchodilators) may elevate intra-abdominal pressure or reduce lower esophageal sphincter tone, thereby promoting reflux.^[29]^ Despite these observations, the causal relationship between the 2 conditions remains uncertain.

In this study, the forward MR analysis treated GERD as the exposure and childhood asthma as the outcome. The IVW method indicated a higher risk of childhood asthma associated with genetically proxied GERD (OR = 1.792; 95% CI, 1.661–1.933; *P* = 2.033 × 10^−51^), demonstrating high statistical significance (*P* < .001). Interpreted on the log-odds scale, this estimate implies that, for a child with a one-unit higher genetically proxied liability to GERD, the odds of childhood asthma are increased by about 79%; for example, if the baseline cumulative risk of childhood asthma were 10%, the corresponding risk would increase to roughly 17% in the presence of such an increase in GERD liability. From a comparative standpoint, the magnitude of this effect is similar to that reported for some established determinants of childhood asthma such as parental asthma, early-life severe viral wheeze, and aeroallergen sensitization (often yielding odds ratios in the range of 1.5–3.0), and exceeds that of several common environmental or lifestyle factors (including passive smoke exposure, traffic-related air pollution, and childhood obesity) which typically confer more modest increases in risk (odds ratios ~1.2–1.5). Taken together, these findings suggest that GERD represents a clinically meaningful, nontrivial contributor to childhood asthma risk from a genetic-instrumental perspective. Estimates from the 4 complementary MR approaches (MR-Egger, WM, SM, and WMo) were concordant with the IVW result, further supporting the robustness of this positive causal effect.

In the reverse MR analysis, which treated childhood asthma as the exposure and GERD as the outcome, the IVW estimate showed no association (OR = 1.029; 95% CI, 0.983–1.078; *P* = .225), and the other 4 MR methods yielded concordant null results. An important question is whether this nonsignificant finding reflects a true absence of causal effect or simply insufficient statistical power. Several features of our study argue against a power limitation as the primary explanation. First, the 29-SNP childhood asthma instrument was strong (*R*^2^ = 0.069; *F* = 1118) and the GERD GWAS included 129,080 cases and 473,524 controls, yielding a very large effective sample size. Second, a post hoc MR power calculation (mRnd), using this *R*^2^, the GERD sample size, and a Bonferroni-corrected α = 0.025, indicated essentially complete power (≈100%) to detect even modest effects of childhood asthma on GERD (e.g., odds ratio ≥ 1.10). Finally, the 95% confidence interval around the point estimate (0.983–1.078) is narrow and centered close to the null. Taken together, these considerations suggest that our null finding is unlikely to be due to inadequate statistical power and instead argue against any moderate-to-large average causal effect of childhood asthma on GERD. Nonetheless, we cannot exclude smaller effects below our detectable threshold, nonlinear relationships, or subtype-specific associations that would require even larger and more finely phenotyped datasets to elucidate.

Innovation of this study. Prior observational evidence has largely come from case–control and cohort designs that reveal correlations between GERD and asthma but cannot establish causality. Such designs are vulnerable to confounding by lifestyle, environmental exposures, and comorbidities, and to reverse causation.^[30,31]^ Here, we used MR, employing genetic variants as IVs to mitigate confounding and reverse causation, thereby strengthening causal inference. We evaluated both directions (GERD → childhood asthma and childhood asthma → GERD) via a bidirectional two-sample MR framework to provide a comprehensive assessment of causality. Compared with traditional observational studies, MR offers more reliable evidence for causal inference.^[32,33]^ We leveraged large-scale GWAS summary data from the OpenGWAS repository to enhance generalizability and credibility of the findings. Finally, we triangulated results across multiple MR estimators (IVW, MR-Egger, WM, SM, and WMo) and conducted extensive diagnostics, which collectively increased the stability and robustness of our conclusions.

This study suggests that, from a genetic standpoint, GERD increases the risk of childhood asthma; prior physiological research also supports a microaspiration-vagally mediated reflex-airway hyperresponsiveness pathway, providing biological plausibility.^[34]^ Beyond mechanical and reflex mechanisms, inflammatory pathways may also contribute to the GERD-asthma link. Reflux-related esophageal epithelial injury can stimulate the release of multiple cytokines and chemokines (e.g., IL-4, IL-5, IL-13, IL-6, IL-8, and TNF-α), which may spill over into the airway compartment, amplify type 2-skewed inflammation, and enhance bronchial hyperresponsiveness and mucus production.^[35]^ Such cytokine-mediated crosstalk between esophageal and airway inflammation provides additional biological plausibility for a causal effect of chronic GERD on childhood asthma, particularly in children with atopic or eosinophilic phenotypes.^[36]^ In addition, extraesophageal phenotypes of GERD (e.g., reflux-related chronic cough) are closely intertwined with airway disease; meta-analytic evidence indicates that surgical anti-reflux interventions confer benefits for refractory reflux-related chronic cough, suggesting that in well-defined populations, controlling reflux may ameliorate airway symptoms.^[37]^

Even if a genetic causal relationship holds, multiple randomized trials have shown that in asthmatic patients without typical reflux symptoms, empiric proton-pump inhibitor (PPI) therapy does not improve asthma control; pediatric studies likewise show no benefit and an increased incidence of adverse events.^[38,39]^ Potential explanations include: MR reflects long-term/cumulative exposure effects, whereas short-term acid suppression is insufficient to reverse established airway remodeling; PPIs suppress acid only and do not address weakly acidic/nonacid reflux or microaspiration; and heterogeneity in pediatric asthma phenotypes, endpoints, and follow-up duration reduces the power of drug trials to detect an effect.^[40]^

Drawing on genetic and clinical evidence, routine empiric use of PPIs solely to improve asthma is not recommended, particularly in the absence of reflux symptoms. For patients with nocturnal symptoms, clinical suspicion of reflux, or refractory phenotypes, guideline-directed objective evaluation (e.g., pH-impedance monitoring) and individualized management are advised. For highly selected populations identified by the evidence, surgical or endoscopic anti-reflux interventions may be considered, but their risk-benefit profile requires rigorous evaluation in pediatric populations.^[41]^

The comorbidity burden among patients with asthma is generally high; a large-sample review in Medicine showed a marked increase in multisystem comorbidities in individuals with asthma.^[42]^ Our results suggest that not all common comorbidities reflect bidirectional causality; in management, it is important to distinguish causal factors, exacerbating factors, and coincident phenomena to avoid overtreatment or misallocation of resources.

This study has several limitations. First, it is based primarily on GWAS conducted in individuals of European ancestry; residual sources of bias may remain, including issues specific to genetic instruments for binary traits, phenotype misclassification, and potential sample overlap between large population-based cohorts. Thus, the causal estimates primarily reflect genetic architecture in European populations and may not be directly generalizable to other ancestries. Second, adiposity is a well-recognized shared risk factor for both GERD and asthma, and residual pleiotropy through body mass index (BMI) or related anthropometric traits cannot be completely excluded. Ideally, multivariable MR including BMI as an additional exposure would help distinguish the direct effect of GERD from pathways mediated or confounded by adiposity. However, in the present study we were unable to implement a robust multivariable MR model because harmonized summary-level BMI data that are clearly independent of both the GERD GWAS and the pediatric asthma GWAS, and age-matched to the childhood asthma phenotype, were not available. Instead, we sought to mitigate adiposity-related pleiotropy by screening candidate GERD instruments using LDtrait, removing variants with strong associations with adiposity and cardiometabolic traits, and applying multiple pleiotropy-robust estimators together with MR-PRESSO and leave-one-out analyses in sensitivity checks. Third, although we quantified instrument strength and performed an MR-specific post hoc power calculation for the reverse direction (childhood asthma → GERD), we did not conduct an a priori, conventional power analysis at the design stage. The available calculations indicate excellent power to detect moderate effects (e.g., odds ratio ≥ 1.10), but smaller or nonlinear effects may still have gone undetected; this should be recognized as a residual limitation, particularly when interpreting the null findings for the reverse pathway. Finally, replication in multi-ancestry cohorts and pediatric, phenotype-specific resources is warranted, alongside formal BMI-adjusted multivariable MR and colocalisation analyses when suitable data become available, as well as clinical studies with longer follow-up to bridge the gap between genetic causality and interventional efficacy.

In summary, this bidirectional MR study is methodologically innovative in leveraging germline genetic variants to minimize confounding and reverse causation. From a genetic epidemiology perspective, we provide robust evidence for a positive causal effect of gastroesophageal reflux on the risk of childhood asthma, whereas there is no evidence for a causal effect in the reverse direction. These findings offer clinically relevant insights and may inform prevention, clinical management, and future research.

## 5. Conclusion

In this bidirectional two-sample MR analysis, genetic evidence supports a positive causal effect of GERD on the risk of childhood asthma, whereas there is no evidence for a causal effect in the reverse direction. Robustness across complementary MR estimators, absence of directional pleiotropy and outliers, and stable leave-one-out profiles strengthen the inference. Clinically, these findings argue against routine empiric proton-pump inhibitor therapy solely to improve asthma control in the absence of reflux symptoms; instead, objective reflux assessment (e.g., pH-impedance) and individualized management should be considered for selected phenotypes. From a methodological standpoint, the study’s strengths include large GWAS datasets and a prespecified primary IVW framework; limitations include the predominance of European-ancestry data, the use of binary phenotypes with possible misclassification, and potential sample overlap. Future work should replicate these results in multi-ancestry pediatric cohorts, integrate multivariable MR and colocalization to address residual confounding, and align clinical trials with biologically relevant endpoints (e.g., microaspiration/nonacid reflux) to bridge the gap between genetic causality and interventional efficacy.

## Acknowledgments

We thank the IEU OpenGWAS project for access to summary-level GWAS data and the LDlink team for providing the LDtrait resource.

## Author contributions

**Methodology:** Lijuan Xiong.

**Project administration:** Lianfu Ding, Lijuan Xiong.

**Resources:** Lijuan Xiong, Qing Li.

**Software:** Lianfu Ding, Lijuan Xiong, Qingfa Chen, Hong Liu, Qing Li.

**Supervision:** Lianfu Ding, Lijuan Xiong, Qingfa Chen, Hong Liu.

**Validation:** Lianfu Ding, Qingfa Chen, Hong Liu, Qing Li.

**Visualization:** Qingfa Chen, Hong Liu.

**Writing – original draft:** Hong Liu.

**Writing – review & editing:** Hong Liu, Qing Li.

## Supplementary Material




