# Supplementary material for: Gastroesophageal reflux disease and childhood asthma: A bidirectional two-sample Mendelian randomization study
Source: Medicine (Baltimore). 2026 Apr 10;105(15):e48171. doi: 10.1097/MD.0000000000048171 (PMC13593192; doi:10.1097/MD.0000000000048171)
Supplement: Supplementary file 1 [file medi-105-e48171-s001.pdf]

Supplementary Table S1. Excluded SNPs and their associations with GERD and childhood asthma

SNP	effect_allele.exposure	other_allele.exposure	eaf.exposure	beta.exposure	se.exposure	pval.exposure	effect_allele.outcome	other_allele.outcome	eaf.outcome	beta.outcome	se.outcome	pval.outcome
rs1011407	G	A	0.121628	-0.0420618	0.00735917	1.09343e-08	G	A	0.0833426	0.0025	0.0143	0.8588
rs10133111	A	G	0.162996	0.0417875	0.00650789	1.35338e-10	A	G	0.232626	0.0003	0.0122	0.9787
rs1021363	G	A	0.641992	-0.0312017	0.00502204	5.09976e-10	G	A	0.686612	0.0021	0.0097	0.8263
rs12357321	A	G	0.311087	0.0317159	0.00523077	1.33325e-09	A	G	0.226976	0.0399	0.0101	7.87898e-05
rs12997558	A	G	0.358751	0.0278184	0.00502214	3.03941e-08	A	G	0.291524	-0.0081	0.0097	0.4019
rs2023878	T	C	0.192377	-0.0362846	0.00611913	3.03501e-09	T	C	0.327038	0.0045	0.0115	0.694201
rs2734839	T	C	0.606693	-0.0283478	0.00492778	8.78537e-09	T	C	0.447657	-0.0353	0.0095	0.000196002
rs2782641	A	G	0.612669	0.0270882	0.00494583	4.32643e-08	A	G	0.652235	-0.0027	0.0095	0.7754
rs3766823	A	G	0.171467	0.0393599	0.00638545	7.09316e-10	A	G	0.179093	0.0572	0.0121	2.40398e-06
rs3828917	T	G	0.041826	0.0671113	0.0120054	2.26945e-08	T	G	0.0318166	-0.0898	0.0235	0.0001366
rs4713692	T	C	0.367808	-0.02761	0.0049863	3.06507e-08	T	C	0.340648	0.0028	0.0095	0.7676

				28	4							
rs569356	G	A	0.140835	-0.037919	0.00690971	4.07005e-08	G	A	0.138787	0.0136	0.0134	0.3092
rs773109	A	G	0.335269	-0.0380572	0.00510213	8.71365e-14	A	G	0.293286	0.0572	0.0098	5.118e-09

Supplementary Table 2. Excluded SNPs and problematic associations

This table lists the GERD-associated SNPs that were excluded during harmonisation and instrument selection in the present Mendelian randomisation analysis. For each variant, we provide the nearest or reported gene/region, a summary of problematic associations reported in previous GWAS/MR studies, and the reason for exclusion from the final instrument set.

SNP	Nearest / reported gene or region	Problematic associations from previous studies	Reason for exclusion in this MR analysis
rs1011407	MIR4432HG locus (chr2)	Large GWAS have reported rs1011407 as associated with body mass index and adiposity-related traits. In GERD-related MR work, this SNP has also been flagged and sometimes removed when adjusting for cardiometabolic confounders.	Because obesity and adiposity are well-established shared risk factors for both GERD and asthma, this SNP was judged to have a high risk of horizontal pleiotropy via adiposity-related pathways and was therefore excluded.
rs10133111	TRAF3 / brain-imaging locus (chr14)	rs10133111 has been associated with brain-imaging phenotypes in schizophrenia and other neuropsychiatric traits. Such traits may influence lifestyle factors (e.g. smoking, medication use, physical activity) that are relevant to GERD and asthma risk.	Given these neuropsychiatric associations and the potential for pleiotropic pathways unrelated to GERD itself, this variant was removed from the instrumental variable set.
rs1021363	SORCS3 (chr10)	Multiple large GWAS have identified rs1021363 in SORCS3 as a genome-wide	Because rs1021363 captures a broad liability to depression-related traits and lifestyle

		significant variant for major depressive disorder and depressive symptoms. Depression is strongly linked to smoking, obesity and cardiometabolic disease, which in turn affect GERD and asthma.	factors that can independently influence childhood asthma, it was considered horizontally pleiotropic and excluded.
rs12357321	Intergenic locus (chr10)	In the present analysis, this variant showed a relatively strong, outlying association with childhood asthma compared with its effect on GERD, behaving as an outlier in influence diagnostics and leave-one-out checks, consistent with outlier-style filtering adopted in previous GERD MR studies.	Because rs12357321 displayed an outlying direct association with childhood asthma that could not be explained solely through GERD, it was treated as a potential horizontal pleiotropic/outlier SNP and removed.
rs12997558	chr2 locus	Phenome-wide scans and anthropometric GWAS have reported rs12997558 to be associated with body fat percentage and adiposity-related measures, and it has also been listed among GERD SNPs identified as outliers in MR-based sensitivity analyses.	As adiposity is a strong shared risk factor for both GERD and asthma, this SNP was considered to have substantial pleiotropic effects via obesity-related pathways and was excluded.
rs2023878	chr19 locus	rs2023878 has been reported as associated	Given its involvement in

		with anthropometric and metabolic traits, including body mass index and related measures, in addition to GERD.	metabolic traits that are common confounders for GERD and asthma, this variant was judged likely to violate the exclusion-restriction assumption and was removed.
rs2734839	DRD2 (chr11)	rs2734839 is a functional DRD2 variant that has been associated with schizophrenia and age at onset of psychosis. Dopaminergic variants influence smoking, antipsychotic use and other behavioural factors relevant to respiratory outcomes.	Because of strong, well-replicated associations with schizophrenia and dopaminergic pathways, rs2734839 was considered likely to affect childhood asthma risk through neurobehavioural mechanisms independent of GERD and was excluded.
rs2782641	PTPRF / chr1 locus	This SNP has been associated with coronary artery disease and cardiometabolic traits in large GWAS, including in patients with type 2 diabetes, and has appeared among GERD variants flagged as pleiotropic outliers in MR studies.	Because cardiovascular and metabolic traits are common confounders for GERD and asthma, rs2782641 was considered to have substantial horizontal pleiotropy and was removed.
rs3766823	chr1 locus	rs3766823 has been reported as associated with whole-body fat mass, waist circumference and	Given its strong links to obesity, which is a shared risk factor for both GERD and asthma, this variant

		other adiposity measures in anthropometric GWAS.	was considered pleiotropic via adiposity-related pathways and excluded.
rs3828917	MICB / HLA region (chr6)	rs3828917 is a GERD locus in the extended HLA region and has been repeatedly flagged as a pleiotropic or outlying SNP in GERD MR studies, including MR analyses of GERD with oral symptoms and autoimmune traits.	Owing to its location in the HLA region and repeated identification as a pleiotropic outlier in prior GERD MR work, rs3828917 was excluded to reduce bias from immune-mediated pleiotropy.
rs4713692	LINC01016 / chr6 locus	rs4713692 is a GERD risk variant that also shows strong associations with platelet traits and autoimmune diseases in GWAS and MR-based phenome scans and has been repeatedly flagged as an outlier in GERD MR analyses.	Because rs4713692 shows broad pleiotropic associations with haematologic and autoimmune traits that may independently influence asthma risk, it was treated as a pleiotropic/outlier SNP and removed.
rs569356	OPRD1 (chr1)	rs569356 is a functional promoter variant in the δ-opioid receptor gene OPRD1. It has been associated with opioid dependence, altered opioid pharmacokinetics and eating or psychiatric disorders, including anorexia nervosa.	Given its strong involvement in addiction- and weight-related pathways, rs569356 was judged likely to have horizontal pleiotropic effects unrelated to GERD alone and was therefore excluded.

		These traits are linked to body weight, addiction and stress-related behaviours that can influence GERD and asthma.	
rs773109	RAB5B / chr12 locus	rs773109 is a GERD risk SNP that also acts as a splicing QTL for RAB5B and has been associated with asthma, eosinophil percentage and other immune traits in GWAS and MR-based scans. It has also been listed among GERD instruments identified as MR-PRESSO outliers.	Because rs773109 appears to influence respiratory and immune phenotypes directly (including asthma) and has been flagged as a pleiotropic outlier, it was considered to violate MR assumptions and was excluded from the final instrument set.

Table S3 SNP-level details for the 67 gastroesophageal reflux disease–associated variants used as instrumental variables in the forward Mendelian randomization analysis (gastroesophageal reflux disease as the exposure and childhood asthma as the outcome).

SNPs	chr	EA	OA	EAF	gastroesophageal reflux			childhood asthma			F
					β	SE	P	β	SE	P	
rs10010963	4	T	C	0.616	-0.027	4.947×10^{-03}	4.918×10^{-08}	-0.015	9.600×10^{-03}	1.250×10^{-01}	29.749
rs10837002	11	G	C	0.351	0.028	5.037×10^{-03}	4.029×10^{-08}	0.015	9.600×10^{-03}	1.218×10^{-01}	30.136
rs11762636	7	A	C	0.180	-0.051	6.256×10^{-03}	1.882×10^{-16}	-0.044	1.190×10^{-02}	2.392×10^{-04}	67.722
rs11953061	5	T	C	0.339	0.028	5.087×10^{-03}	3.099×10^{-08}	0.011	9.800×10^{-03}	$2.522\times10\text{-}^{01}$	30.644
rs12204714	6	T	C	0.632	-0.029	4.994×10^{-03}	7.924×10^{-09}	-0.027	9.600×10^{-03}	5.375×10^{-03}	33.294
rs12453010	17	T	C	0.395	0.030	4.933×10^{-03}	1.746×10^{-09}	0.014	9.400×10^{-03}	1.429×10^{-01}	36.238
rs12598916	16	G	C	0.275	-0.033	5.392×10^{-03}	6.873×10^{-10}	-0.006	1.030×10^{-02}	5.613×10^{-01}	38.056
rs12967855	18	G	A	0.670	-0.037	5.134×10^{-03}	1.088×10^{-12}	-0.019	9.800×10^{-03}	5.532×10^{-02}	50.679
rs13107325	4	T	C	0.074	0.070	9.183×10^{-03}	2.199×10^{-14}	0.078	1.800×10^{-02}	1.334×10^{-05}	58.345
rs1334297	13	A	G	0.734	-0.039	5.455×10^{-03}	1.141×10^{-12}	-0.030	1.050×10^{-02}	4.330×10^{-03}	50.585
rs13409451	2	G	A	0.392	-0.028	4.932×10^{-03}	1.929×10^{-08}	-0.027	9.600×10^{-03}	4.722×10^{-03}	31.564
rs1431196	18	G	A	0.428	0.032	4.864×10^{-03}	2.655×10^{-11}	0.023	9.400×10^{-03}	1.603×10^{-02}	44.417
rs1479405	12	T	C	0.322	0.031	5.151×10^{-03}	9.852×10^{-10}	0.016	9.900×10^{-03}	9.815×10^{-02}	37.354
rs1510719	4	C	T	0.383	-0.039	4.947×10^{-03}	3.836×10^{-15}	-0.016	9.500×10^{-03}	8.630×10^{-02}	61.782
rs1592757	5	C	G	0.356	0.031	5.025×10^{-03}	5.998×10^{-10}	0.025	9.600×10^{-03}	1.040×10^{-02}	38.322
rs1596747	2	G	A	0.494	0.031	4.807×10^{-03}	1.003×10^{-10}	0.005	9.200×10^{-03}	5.654×10^{-01}	41.815
rs1716171	12	T	C	0.790	0.038	5.904×10^{-03}	7.825×10^{-11}	0.041	1.140×10^{-02}	3.551×10^{-04}	42.301
rs17379561	1	T	A	0.144	0.053	6.866×10^{-03}	1.076×10^{-14}	0.035	1.310×10^{-02}	6.751×10^{-03}	59.751
rs1883842	20	G	T	0.279	0.031	5.368×10^{-03}	9.268×10^{-09}	0.010	1.040×10^{-02}	3.532×10^{-01}	32.989
rs1937450	1	G	T	0.538	0.032	4.845×10^{-03}	7.068×10^{-11}	0.008	9.300×10^{-03}	4.190×10^{-01}	42.500
rs2016933	3	G	C	0.730	-0.031	5.421×10^{-03}	1.043×10^{-08}	-0.017	1.050×10^{-02}	1.093×10^{-01}	32.760
rs2043539	7	A	G	0.419	0.027	4.865×10^{-03}	2.240×10^{-08}	0.017	9.300×10^{-03}	7.027×10^{-02}	31.274
rs2106353	7	T	G	0.231	0.037	5.725×10^{-03}	1.372×10^{-10}	0.010	1.130×10^{-02}	3.978×10^{-01}	41.204
rs2145318	6	A	T	0.487	0.035	4.811×10^{-03}	2.028×10^{-13}	0.006	9.500×10^{-03}	5.235×10^{-01}	53.977
rs215614	7	A	G	0.630	-0.033	4.977×10^{-03}	4.084×10^{-11}	-0.002	9.600×10^{-03}	8.597×10^{-01}	43.573
rs2164300	4	T	C	0.523	-0.026	4.827×10^{-03}	4.134×10^{-08}	-0.023	9.300×10^{-03}	1.578×10^{-02}	30.086
rs2240326	3	A	G	0.474	-0.047	4.813×10^{-03}	1.132×10^{-22}	-0.024	9.400×10^{-03}	9.467×10^{-03}	96.029
rs2358016	2	G	C	0.498	0.028	4.810×10^{-03}	4.172×10^{-09}	-0.002	9.200×10^{-03}	7.967×10^{-01}	34.542
rs2396133	7	G	A	0.475	0.029	4.818×10^{-03}	1.109×10^{-09}	0.025	9.200×10^{-03}	8.097×10^{-03}	37.123
rs2396766	7	A	G	0.473	0.032	4.819×10^{-03}	2.335×10^{-11}	0.020	9.300×10^{-03}	3.151×10^{-02}	44.667
rs2744961	6	T	C	0.358	0.029	5.015×10^{-03}	5.811×10^{-09}	0.011	9.600×10^{-03}	2.734×10^{-01}	33.897
rs2815749	1	G	A	0.801	0.039	6.022×10^{-03}	1.074×10^{-10}	0.042	1.170×10^{-02}	2.896×10^{-04}	41.683
rs2834005	21	C	T	0.315	0.030	5.173×10^{-03}	9.420×10^{-09}	0.016	1.000×10^{-02}	1.072×10^{-01}	32.957
rs2838771	21	C	G	0.647	-0.028	5.066×10^{-03}	2.910×10^{-08}	-0.026	9.700×10^{-03}	7.122×10^{-03}	30.767
rs324769	12	T	C	0.449	-0.027	4.833×10^{-03}	3.048×10^{-08}	-0.013	9.300×10^{-03}	1.527×10^{-01}	30.677
rs329122	5	A	G	0.420	-0.029	4.884×10^{-03}	3.055×10^{-09}	-0.022	9.300×10^{-03}	1.711×10^{-02}	35.149
rs3793577	9	G	A	0.538	0.027	4.850×10^{-03}	2.494×10^{-08}	0.021	9.300×10^{-03}	2.358×10^{-02}	31.066
rs3863241	8	T	C	0.527	0.032	4.815×10^{-03}	1.488×10^{-11}	0.020	9.300×10^{-03}	3.416×10^{-02}	45.550
rs4300861	2	T	C	0.382	0.031	4.949×10^{-03}	5.431×10^{-10}	0.022	9.500×10^{-03}	2.260×10^{-02}	38.516

rs4382592	9	G	T	0.700	-0.030	5.251×10^{-03}	8.199×10^{-09}	-0.014	1.000×10^{-02}	1.537×10^{-01}	33.227
rs6711584	2	A	G	0.452	0.032	4.840×10^{-03}	2.656×10^{-11}	0.012	9.400×10^{-03}	2.024×10^{-01}	44.416
rs6722661	2	A	G	0.365	-0.032	5.004×10^{-03}	1.146×10^{-10}	-0.022	9.600×10^{-03}	2.403×10^{-02}	41.555
rs6780459	3	T	A	0.747	0.031	5.521×10^{-03}	3.140×10^{-08}	0.009	1.060×10^{-02}	4.016×10^{-01}	30.619
rs7032155	9	A	C	0.592	0.028	4.914×10^{-03}	1.627×10^{-08}	0.031	9.400×10^{-03}	8.487×10^{-04}	31.896
rs7206608	16	G	C	0.323	0.029	5.145×10^{-03}	1.458×10^{-08}	0.017	9.900×10^{-03}	7.976×10^{-02}	32.109
rs7241572	18	A	G	0.209	0.037	5.975×10^{-03}	9.493×10^{-10}	0.044	1.150×10-02	1.310×10^{-04}	37.426
rs7527682	1	G	A	0.537	-0.027	4.822×10^{-03}	3.128×10^{-08}	-0.009	9.200×10^{-03}	3.535×10^{-01}	30.626
rs7541875	1	G	A	0.426	0.027	4.850×10^{-03}	1.612×10^{-08}	0.031	9.300×10^{-03}	7.758×10^{-04}	31.913
rs7600261	2	T	C	0.306	0.034	5.221×10^{-03}	9.473×10^{-11}	0.003	1.010×10^{-02}	7.497×10^{-01}	41.927
rs7612999	3	A	G	0.245	0.031	5.595×10^{-03}	4.898×10^{-08}	0.004	1.080×10^{-02}	7.107×10^{-01}	29.757
rs761777	10	G	A	0.254	0.035	5.545×10^{-03}	4.715×10^{-10}	0.015	1.060×10^{-02}	1.728×10^{-01}	38.792
rs7675588	4	A	C	0.795	-0.034	5.954×10^{-03}	1.803×10^{-08}	-0.017	1.150×10^{-02}	1.474×10^{-01}	31.696
rs7685686	4	G	A	0.422	-0.028	4.892×10^{-03}	1.144×10^{-08}	-0.010	9.300×10^{-03}	2.879×10^{-01}	32.579
rs7942368	11	T	C	0.215	-0.034	5.919×10^{-03}	9.541×10^{-09}	-0.020	1.140×10^{-02}	$7.469\times1^{0-02}$	32.933
rs903678	1	A	G	0.339	0.028	5.085×10^{-03}	4.891×10^{-08}	0.008	9.800×10^{-03}	4.464×10^{-01}	29.759
rs903959	8	A	T	0.399	0.029	4.916×10^{-03}	2.989×10^{-09}	0.026	9.500×10^{-03}	6.592×10^{-03}	35.191
rs9372625	6	A	G	0.383	-0.038	4.954×10^{-03}	2.618×10^{-14}	-0.019	9.500×10^{-03}	4.477×10^{-02}	58.002
rs9373363	6	G	A	0.254	-0.033	5.560×10^{-03}	4.135×10^{-09}	-0.025	1.060×10^{-02}	1.769×10^{-02}	34.559
rs9396740	6	A	G	0.249	-0.031	5.559×10^{-03}	1.466×10^{-08}	-0.033	1.070×10^{-02}	1.882×10^{-03}	32.098
rs942065	14	A	G	0.634	0.031	5.009×10^{-03}	8.446×10^{-10}	0.024	9.600×10^{-03}	1.183×10^{-02}	37.655
rs9517313	13	C	G	0.383	0.033	4.941×10^{-03}	2.048×10^{-11}	0.017	9.400×10^{-03}	7.057×10^{-02}	44.925
rs9529055	13	A	G	0.476	0.027	4.816×10^{-03}	3.105×10^{-08}	0.005	9.200×10^{-03}	6.038×10^{-01}	30.640
rs9542729	13	G	C	0.202	-0.036	5.999×10^{-03}	1.412×10^{-09}	-0.012	1.140×10^{-02}	2.762×10^{-01}	36.652
rs957345	14	G	C	0.540	0.029	4.824×10^{-03}	1.716×10^{-09}	0.018	9.200×10^{-03}	5.094×10^{-02}	36.272
rs9615905	22	T	C	0.458	0.028	4.838×10^{-03}	1.212×10^{-08}	0.006	9.300×10^{-03}	5.278×10^{-01}	32.467
rs9636202	19	A	G	0.267	-0.035	5.472×10^{-03}	1.510×10^{-10}	-0.002	1.040×10^{-02}	8.176×10^{-01}	41.016
rs9940128	16	A	G	0.422	0.033	4.863×10^{-03}	8.057×10^{-12}	0.018	9.300×10^{-03}	5.874×10^{-02}	46.752
