# Supplementary material for: Gastroesophageal reflux disease and childhood asthma: A bidirectional two-sample Mendelian randomization study
Source: Medicine (Baltimore). 2026 Apr 10;105(15):e48171. doi: 10.1097/MD.0000000000048171 (PMC13593192; doi:10.1097/MD.0000000000048171)

Supplementary Figure S1. Graphical abstract of the bidirectional two-sampleMendelian randomization (MR) workflow and key findings for GERD and childhood asthma

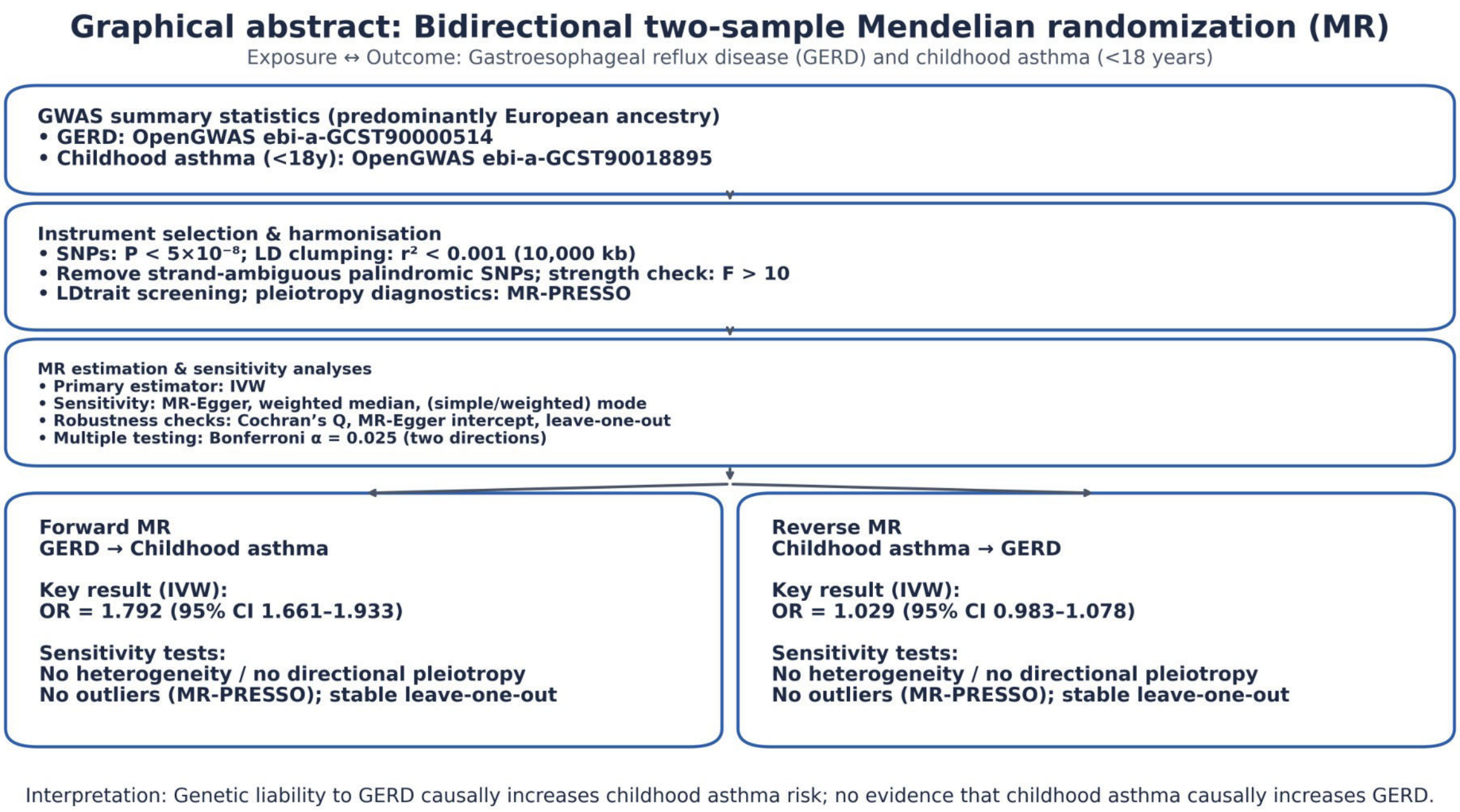

Supplement: Supplementary file 2 [file medi-105-e48171-s002.pdf]
